# A qualitative study of treatment-seeking heroin users in contemporary China

**DOI:** 10.1186/s13722-015-0044-3

**Published:** 2015-11-04

**Authors:** Anna Lembke, Niushen Zhang

**Affiliations:** Department of Psychiatry and Behavioral Sciences, Stanford University School of Medicine, 401 Quarry Road, Stanford, CA 94306 USA; Department of Neurology, Stanford University School of Medicine, Stanford, CA 94306 USA

**Keywords:** China, Heroin, Addiction, Treatment, Methadone, Qualitative

## Abstract

**Background:**

Heroin has emerged as the primary drug of concern in China, with as many as three million contemporary users. Once a Chinese citizen has been identified by Chinese law enforcement as a ‘drug addict’, that individual is ‘registered’ in an official government tracking system for the rest of his or her life, independent of verified rehabilitation and recovery. Most of what is known about heroin users in China is based on studies of registered heroin users participating, often involuntarily, in government-sponsored treatment.

**Methods:**

Using Grounded Theory Methodology, we collected and analyzed in-depth interviews of heroin users voluntarily seeking treatment at a new, non-government-sponsored, for-profit, addiction treatment hospital in Beijing, China.

**Results:**

We identified three major themes among our participants: (1) intense social stigma towards individuals with drug addiction; (2) a desire for anonymous, confidential treatment to avoid social stigma and the loss of personal freedom that accompanies participation in government-sponsored treatment; and (3) a deep mistrust of government-sponsored treatment and a search for more effective alternatives.

**Conclusion:**

Despite a desire for treatment, our subjects were reluctant to access government-sponsored treatment facilities because of fear of a stigmatized identity, fear of loss of personal freedom, and lack of faith in the efficacy and safety of government-sponsored treatments. Their willingness to pay cash at a new, non-government-sponsored, addiction treatment facility illustrates the lengths to which they will go to remain ‘unregistered’ and to discover better alternatives. That the Chinese government allows such facilities to operate outside of government surveillance suggests a new openness to alternative options to combat China’s rising drug epidemic. The efficacy of these alternative options, however, remains in question.

## Background

Beginning in the mid-nineteenth century when Britain defeated the Qing government and forced legal importation of opium (the Opium Wars), opioid use began to escalate in China. By 1906, about 27 % of the adult, male population was addicted to opium [1]. Beginning in the 1950’s, the newly formed Communist government launched a massive, three-year, antidrug campaign, which led to the shutdown of production sites, sentencing of drug offenders, mandatory treatment and rehabilitation of millions of drug addicted persons, and ultimately to the claim that there was a near disappearance of opioid use and drug trafficking [2]. With the establishment of the “open-door” policy in the 1980’s and China’s proximity to major production sources (the Golden Triangle and the Golden Crescent), illegal drug trafficking re-emerged in China [1].

Heroin is now the primary drug of concern in China, followed by amphetamine-type stimulants and tranquilizers [3]. The Chinese Ministry of Public Security 2012 Annual Report on Drug Control estimates that 1.156 million of the 1.794 million drug-dependent persons registered by the national public security system are addicted to heroin [4]. By some earlier estimates, the actual number of heroin users, including the unregistered users, is more than twice that number [3]; and the total number of drug users in China may be as high as 7 million, with China predicted to have the most heroin users of any country in the world within 5 years [5].

To date, the Chinese government has set up three types of facilities to target the problem of drug use and addiction. They include the compulsory detoxification institutions (CDI), the rehabilitation units through labor (RUL), and the voluntary detoxification institutions (VDI) such as methadone maintenance treatment (MMT) clinics. The CDI and RUL are run by law enforcement agencies and the MMT is run by the Department of Health [6].

Currently, the regulations stipulate three levels of penalty for illicit drug use: (1) First time illicit drug users are fined and/or placed in local detention centers for up to 2 weeks; (2) regular illicit drug users and those with drug dependencies are confined to residential rehabilitation centers for periods from one to 6 months; (3) drug users who relapse after compulsory detoxification are placed in compulsory labor re-education centers for one to 3 years [6, 7]. Once an individual has been identified by law enforcement agencies as an illicit drug user, that individual is ‘registered’ in official government tracking systems for the rest of his or her life, independent of verified rehabilitation and recovery. Despite these measures, the number of registered drug users in China increased 19-fold between 1990 and 2009 [8].

The first MMT clinics were opened in 2004 [9] as a reaction to the failure of the strictly punitive approach that was stated in the “Regulations on Prohibition against Narcotics” from 1990 [6]. In 2004, eight MMT clinics served 1029 drug users. By the end of 2011, MMT clinics had expanded to 738 clinics serving 344,254 drug users. Now MMT reaches approximately 30 % of registered intravenous drug users in China [9]. According to a systematic review by Zhang et al., MMT services in China are closely monitored by the public security, and police raids and arrests near MMT sites are common. Furthermore, random urine testing in MMT clinics that are positive for heroin can result in immediate compulsory detention. Police arrest and detention in compulsory rehabilitation is the most common cause of drop out (22.2 %) [9].

The majority of the research on heroin users in China to date is based on studies of registered users in government treatment facilities for compulsory detoxification and rehabilitation. Findings show that most are younger than 35 years, have lower levels of education (high school or less), have a history of alcohol and tobacco use prior to drug use, have unstable jobs, and have a high likelihood of relapse [2, 6].

In this explorative, qualitative study, we collected and analyzed in-depth interviews with heroin users seeking treatment for heroin addiction at a new, voluntary, for-profit, addiction treatment facility in Beijing, China, which is government-approved but not government sponsored. Its clients are not required to disclose their identity or become registered with the government tracking system. Using Grounded Theory Methodology, we sought to capture a deeper understanding of the precipitants, experiences, and beliefs among contemporary heroin users living in China who are voluntarily seeking treatment at a non-government-sponsored treatment facility.

## Methods

### Research setting

All interviews were conducted at a voluntary, non-government sponsored, inpatient, addiction treatment hospital in Beijing, China, which will henceforth, pseudonymously, be called “New Hospital”. We chose New Hospital as it was the only treatment facility in Beijing which granted us permission to interview patients in person. We solicited several government-sponsored treatment facilities in Beijing in the months preceding field work, but despite our efforts, we were not granted permission.

New Hospital officially opened on August 2013 as a for-profit, private, civilian-invested venture (“ming ban qi ye”) as distinct from more traditional government-run institutions. Although Beijing government officials are aware of the existence of New Hospital and have sanctioned its existence, as attested to by an official document we were shown, patients at New Hospital can receive their treatment without triggering registration with government officials as an illicit drug user, a status which becomes permanently attached to the individual identification card in the national civilian database.

New Hospital has 40 inpatient beds dedicated to the treatment of drug addiction, and averages 20–40 admissions per month. The average length of stay is 1 week. Eighty percent of patients seek treatment for heroin addiction. Ten percent seek treatment for methamphetamine addiction. Ten percent seek treatment for other drug addictions, including most commonly tramadol and meperidine. In terms of the patient population at New Hospital, the majority (75 %) of patients comes from outside the Beijing area, predominantly from urban areas in the provinces of Inner Mongolia and ShanXi. Over 90 % of admitted patients are men, and most are in their 40’s. The estimated percent of patient who returned at least once in the first year for a second round of treatment was approximately 40–50 %.

New Hospital has a website describing its services, but otherwise relies primarily on word-of-mouth referral from patients and providers. The advertised treatments offered at New Hospital include methadone detoxification, adjunctive psychiatric treatment including psychiatric medications, and behavioral interventions.

New Hospital does not take health insurance. Anyone who seeks admission and has the resources to pay out of pocket for the treatment can be admitted to New Hospital. Patients are expected to pay cash up front depending on the number of days they plan to stay. The average cost per day for treatment is 300 renminbi (RMB), or fifty dollars, not including meals, but can cost more or less depending on a pre-ordained algorithm that includes severity of drug use history, type of drug addiction, private vs shared room, etc. To put that in perspective, an average salary in Beijing, China is 5000 RMB per month [10], or 833 US dollars. Given that the average length of stay is 1 week, treatment at New Hospital costs on average 2100 RMB, or nearly half of the average monthly salary.

Two psychiatrists staff the unit at any given time, along with a total of eight ancillary health care providers. Providers are paid according to a fee structure that is at least partly based on the number of patients seen, and the type of care delivered. In other words, New Hospital runs on a cash-basis fee-for-service business model similar to many US hospitals.

### Data collection and analysis

Approval was obtained from the Stanford Institutional Review Board (IRB) to conduct anonymous interviews with patients with addiction in China. The consent form was translated into Mandarin Chinese and approved by the Stanford IRB in both English and Mandarin. The study protocol and consent form were subsequently approved by the administration of New Hospital prior to recruitment.

In July 2014, we conducted nine, in-depth interviews of heroin users with self-identified ‘heroin addiction’ seeking treatment for such at New Hospital. The sample was primarily determined by opportunity and access, which serendipitously included five unregistered heroin addicted persons (55.5 % of the study sample), an otherwise difficult population to capture. The criteria for participation in the study were: (1) Seeking treatment for heroin use/addiction, and (2) willingness to undergo an in-depth interview. All patients met DSM-V criteria for an opioid use disorder, although that was not an inclusion criterion per se.

Participants were recruited by New Hospital staff on the day of the interview, by asking who might be willing to sit for an interview. A portion of potential participants declined to participate, but specific number of refusals was not obtained. In a system known to be coercive, the knowledge of refusal of participation suggests true voluntariness, which is of ethical importance. Those who agreed to participate were led to a private room separate from regular hospital rooms and corridors, to maximize privacy. Prior to initiating the interview, the interviewer reviewed the verbal consent form with the potential participant. The consent form detailed the study purpose, procedures, and potential risks. Potential participants were again informed that participation was voluntary. None refused to participate at that juncture. Consent was obtained verbally. At no point was the participant asked his/her name. Nor was the participant’s name associated with the written notes at any time in the course of obtaining data, including on the verbal consent form, which did not include a signature line.

The research team consisted of the principal investigator who served as the interviewer. She was also the first reviewer for coding of the transcripts. The principal investigator is a psychiatrist and addiction medicine expert trained in qualitative interviewing skills, ethics, and safety. The interviews were conducted in Mandarin Chinese with the assistance of a skilled interpreter who had experience as a medical translator. The principal investigator asked questions in English and the interpreter translated the questions into Chinese. The interpreter then translated the participants’ responses into English. The principal investigator took hand-written notes in English during the interview, which later served as transcripts for coding. The research team also consisted of an independent reviewer who also coded the transcript and analyzed the data. The independent reviewer is a neurologist trained in qualitative interviewing skills, ethics, and safety.

This study used Grounded Theory methodology [11, 12]. Grounded Theory seeks to characterize complex social phenomenon by inductive analysis of data which are gathered in an iterative process, which allows multiple potential hypotheses to emerge in the course of data collection, instead of generating hypotheses a priori. Subsequent interviews seek to challenge or confirm emerging themes. Interviews are concluded when the coders identify that emerging themes have been saturated.

This study represents a convenience sample of those individuals at New Hospital who were both willing and able to be interviewed during the time frame of the study period. Despite the small sample size (N = 9), both reviewers concurred that the sample reached saturation in terms of the types of themes that emerged from the interviews.

The in-depth interviews lasted up to 120 min. The interviewer began simply by asking participants to describe how the events of their lives led them to seek treatment at New Hospital. As per Grounded-Theory Methodology, the interviewer sought to minimize undue influence on the natural unfolding of the autobiographical narratives. The interviewer might interject at points to clarify or explore some point the participant raised in more depth; but with intention, the content generated was initially participant-driven. Two to three interviews were conducted during the morning and analysis was performed immediately in the afternoon (the same day as the interview) by the first and second reviewer. Coding was performed by each reviewer independently. Data were broken down into smaller components and labeled by topic such as “stigma” and “forced labor camps.” Qualitative coding software was not used.

After coding was completed, the two reviewers compared coding lists to understand and explain variation in the data [13]. The codes were then combined and related to one another and classified into emerging themes/concepts such as “a desire for anonymous, confidential treatment.” Analysis highlighted relationships and demonstrated gaps in the existing data. For example, we wondered why individuals who were already in the government registry would value anonymous, confidential treatment, when their anonymity had already been compromised. Questions for future interviews were augmented to fill in those gaps and guide deeper exploration of the emerging theory. Theoretical saturation was reached when all of the major themes were well developed and supported by data.

When all the interviews were complete, the two independent reviewers came to consensus on the most important themes that emerged from the data. These themes constitute the results of this qualitative work (substantive theory), and are described below. Any text in quotes is a verbatim quote from a participant.

## Results

### Description of study sample

The sample of participants included six men and three women. Two were between the ages of 30–39 and seven were in their 40s. Four of the participants were from Inner Mongolia, one was from Beijing, three were from Tianjing, and one was from Shanxi. Only one of the participants had attended college and eight had not finished high school. Five were married, three were divorced, and one was single. Almost all (8/9) had children. Most (7/9) of the participants were either employed or self-employed. Seven reported using heroin for greater than a decade. The age of first use varied widely, from teens to 40’s. Six subjects reported using heroin through inhalation (smoking, snorting, ‘chasing the dragon’) and three administered heroin intravenously.

Four of the subjects reported spending some time within a compulsory detoxification centers (rehabilitation unit through labor RUL), and these individuals were also registered with the government as ‘illicit drug addicts’. The other five were unregistered. Only one of the participants had received treatment through an official government-sanctioned methadone maintenance clinic (MMT). All New Hospital patients reported methadone-facilitated detoxification as the mainstay of their treatment at New Hospital. All participants had co-morbid tobacco use, seven used alcohol on a regular basis, and six had used other illicit drugs at some point in their lives after trying heroin first.

### Theme #1: Intense social stigma towards individuals with drug addiction

All subjects universally endorsed severe stigma towards drug-addicted persons in China. They described experiences of being ostracized and shunned, or fear of such, once their drug use became known to others. Furthermore, registered users described little hope of establishing a new, non-stigmatized, non-drug using identity, even after sustained abstinence.

“Chinese people look down on drug addicts. Heroin addicts have … a lot of loneliness. People don’t understand what a drug addict feels.” (Male, 46 years old, never married, a registered heroin user.)

“No way can I tell my husband or my daughter about my addiction. They would never be able to accept it. My daughter is 17. She’s in America now. She is self-confident and has a bright future. This would destroy her.” (Female, 40 years old, married, an unregistered heroin user.)

“My friends don’t know I’m addicted, but if they did, they would not want to have anything to do with me.” (Female, 45 years old, married, an unregistered heroin user.)

“As a drug addict, society abandons you. It’s hard to get on the right path. People still don’t treat you like a real person. Even once you’ve quit, you’re still viewed as a drug addict and that becomes your identity. No one outside my circle of drug friends wants to associate with me, but I can’t associate with my old friends or I will relapse. I want to work, but no one will hire me. I’ve become a total waste of a person. I feel so much regret. It has destroyed my family and my life. Addicts are people too. Not all addicts are bad people. They are good people too.” (Male, 44 years old, divorced, a registered heroin user.)

### Theme #2: A desire for anonymous, confidential treatment to avoid social stigma and loss of personal freedom that accompanies participation in government-sponsored treatment

All of our unregistered subjects (5/9) endorsed choosing New Hospital because it provided an anonymous alternative to government-sponsored treatment options, allowing them to get treatment without being identified on the public registry as ‘drug addict’. They viewed becoming registered, and the ensuing social stigma, as affecting not just their lives, but also their children’s lives.

“Part of the reason I relapsed is because I don’t have methadone. I thought about buying methadone from the black market, but it’s too expensive -2,000 RMB for 500 ml. So, instead, I just buy heroin. I don’t dare go to a methadone clinic because I don’t want my name on the public record.” (Male, 38 years old, married, an unregistered heroin user.)

“Once you go to an MMT clinic, you’re on the record, and if you don’t show up, the government will come check up on you.” (Female, 45 years old, married, an unregistered heroin user.)

“I would not go to a methadone maintenance clinic because it would ruin my business. In China, they have a tough view of addicts and drug use is a crime. In China they don’t trust addicts. Chinese people believe you can’t do business with addicts.” (Female, 40 years old, married, an unregistered heroin user.)

“If my name gets on the public record, my children can’t join the armed forces and they can’t ever get a government job.” (Male, 38 years old, married, an unregistered heroin user.)

Based on the reports of registered users in our sample (4/9), the fears of unregistered users were well-founded. Some registered users described government surveillance and loss of personal privacy, even in the absence of participation in government treatment. Government authorities visited them in their homes un-announced to ask them about drug use, and sometimes demanded urine toxicology tests on the spot to assess their use. Their neighbors learned what the visits from authorities were for, and soon everyone they had contact with—neighbors, employers, remote acquaintances—were aware of their drug user status. One subject described relocating to a new town to avoid the discrimination she experienced in her home neighborhood.

Registered users chose New Hospital over government-sponsored treatments to avoid the loss of personal freedom they risked if their ongoing heroin use was detected while participating in voluntary treatment. They had each experienced China’s rehabilitation units through labor (RULs) first hand, and described these places as punitive and ultimately ineffective.

“I spent 6 months in a forced program in Inner Mongolia. It was too horrifying. I was treated like less than an animal. I had no human rights. I can’t even talk about it.” (Male, 44 years old, divorced, a registered heroin user.)

“In 9/2012 I was arrested for heroin and that was when my family found out about my addiction. I went to a forced detention center for seven months and it was basically like jail. There was no medicine, no methadone, no freedom. They taught us about detention center regulations and Chinese laws regarding drug use. They had us watch videos of people getting arrested for drugs. I talked to my family through a glass window. I made some friends. I got discharged early to care for aging parents, and because I wasn’t having any more withdrawal. The loss of freedom made everything painful. And I swore to myself that I would never do drugs again. One month later, I relapsed.” (Male, 46 years old, college graduate, never married, a registered heroin user.)

“I went to a camp in Datong. I stayed for two years and three months. They made us work sometimes, but there wasn’t much to do. Sometimes we made paper bags. There was no treatment in the camp, no methadone, no detox, no medical follow-up after I was discharged from the camp. I relapsed after 20 days.” (Male, 41 years old, middle school graduate, divorced, a registered heroin user)

“I was arrested for heroin and spent six months in a forced detention camp. It was like jail. We lived 20-30 people to a cell. If there was work, we did it, like making plastic flowers. Otherwise we would just sit there and do nothing. No medication, no treatment. I relapsed 6 months after I got out.” (Female, 46 years old, divorced and remarried, a registered heroin user.)

### Theme #3: A deep mistrust of government-sponsored treatment, and a search for new, more effective alternatives

Among all of our participants, but particularly among registered users who had participated in government-sponsored addiction treatment, there was a deep sense of mistrust and futility regarding government-sponsored treatments. Many were skeptical of methadone maintenance in principle.

“I tried methadone maintenance for 6 months. But they never gave me enough. To feel better, I would have to shoot up heroin at the same time. They don’t really care if you get better or not.” (Male, 44 years old, married, a registered heroin user.)

“I’ve never been on methadone maintenance, and I won’t try it. It’s more addictive than heroin. Methadone must be worse than illegal drugs, otherwise why would the government control it so closely?” (Male, 41 years old, divorced, a registered heroin user)

“I am not interested in methadone maintenance. Methadone is more addictive than heroin. My boyfriend was on methadone maintenance for a while, and was using heroin at the same time, so I know it doesn’t work.” (Female, 46 years old, divorced, a registered heroin user.)

Subjects expressed hope that New Hospital, because it was new, might provide more effective and innovative solutions. For most, New Hospital was just one more stop in a long line of treatments, and many were returning to New Hospital a second, third, and in one case a sixth time.

“My Mom brought me here, and I came because I felt sorry for her. She hopes that since this is a new hospital, they will be able to help me. I want to stop. From my heart I do. But I’ve tried to kick the habit for 10 years, then one taste gets me back to the beginning again.” (Male, 41 years old, divorced, a registered heroin user.)

“I came here for the psychiatric treatment they advertise on their website, along with the addiction treatment. But I haven’t gotten any psychiatric treatment. There are no activities. Just a methadone taper. That’s it. There is not anywhere to exercise either. Sometimes I can’t sit still. I just pace in my room and in the hallway. I can’t even go outside.” (Male, 38 years old, married, an unregistered heroin user.)

One subject received “addiction surgery” at another private facility before coming to New Hospital, a ‘surgery’ we presume involved an opioid-antagonist implant.

“In 2007, I got the addiction surgery. I went to Wuhan province for the surgery. My parents made me go, and they paid for it. After the surgery, I tried shooting up heroin and I couldn’t get the feeling. For the next 6 months I shot up everyday with no feeling. I did not think about stopping because I still had money. After six months, the feeling came back. I guess the surgery didn’t work, so I’m here now, hoping they’ll have something new and better for me.”(Male, 38 years old, married, an unregistered heroin user.)

## Discussion

We explore the beliefs and motivations of a subset of treatment-seeking heroin users in contemporary China; specifically, a small group of individuals willing to pay as much as half of their monthly earnings on a week’s worth of treatment at New Hospital. New Hospital is a private, for-profit, non-government-sponsored, inpatient, addiction treatment setting offering methadone detoxification as the main-stay of its treatment for clients with opioid-use disorders.

In China, the existence of New Hospital, outside of government surveillance and with a regular base of customers, is remarkable given that methadone treatment is offered *for free* at government-sponsored treatment facilities throughout the country. Our data suggest that New Hospital and facilities like it have emerged in China’s current addiction-treatment-landscape to serve the needs of those wanting treatment, but wishing to avoid the stigmatized identity and loss of personal freedom that comes with participating in government-sponsored treatment.

Both registered and unregistered heroin users in our study had personally experienced and/or were aware of intense social stigma directed toward heroin users in China. Our findings are consistent with several studies exploring stigma and addiction in China. In a survey study of beliefs about persons with drug addiction in China, most respondents endorsed believing that drug addiction is caused by a person’s own weak will (82.0 %) and hedonistic lifestyle (81.3 %) [14]. Survey respondents furthermore expressed a strong desire to have limited or no contact with heroin users. Another study found that Chinese employers discriminate against applicants with substance use disorders [15]. According to a review by Tang and Hao, drug addiction, along with prostitution and gambling, in most government documents, is referred to as an ‘ugly social phenomenon’ and a personality flaw, rather than a medical disorder; and both lay persons and professionals think punishment is an important component of drug treatment [16].

According to our data, unregistered heroin users seeking treatment at New Hospital are motivated by a desire to preserve their anonymity and to avoid the social, legal, and economic consequences of becoming registered with the government as ‘drug addicts’. At least one prior study of heroin users in China has identified fear of government registry as a barrier to participation in government-sponsored methadone maintenance clinics, with unregistered participants expressing a unanimous desire to avoid any treatment that might confer a status of ‘illicit-drug user’ with the Chinese authorities [17].

Registered users seeking treatment at New Hospital sought confidential treatment as well, to avoid the threat of imprisonment at forced detention camps, where they had experienced a demeaning loss of personal freedom, received little or no addiction treatment, and by most accounts, engaged in little labor. In 1999, about 224,000 drug-addicted individuals resided at compulsory detoxification centers and 120,000 drug-addicted individuals resided at re-education-through-labor institutes [16]. To date, there are sparse data on the living conditions and activities inside these compulsory detoxification and labor re-education centers. According to Amon et al., “These centers are neither prisons nor hospitals: individuals are held without due process protections or judicial oversight of detention. At the same time, the centers lack evidence-based drug-dependency treatment and, often, any trained health care personnel” [18].

Many of our subjects expressed a desire for new and innovative treatments as another prime motivator for seeking help at New Hospital. Whether for-profit ‘rehabs’ like New Hospital actually provide innovative or even effective treatment, remains to be seen. At least one participant expressed skepticism about New Hospital’s mission and integrity, suggesting it may not provide what it promises, and as such, may be just one more for-profit enterprise in the burgeoning Chinese capitalistic economy, preying on the desperate and vulnerable. Regardless, the coexistence of New Hospital alongside government-sponsored treatment, implies a new openness on the part of the government to augment the current system, possibly as part of a broader campaign to curb China’s rising drug problem.

China’s current system of registering and imprisoning drug-addicted persons is not unique to China. Vietnam has a similar system in which addicted persons are registered and monitored using a national database. Since the 1990‘s, the internment of drug users in compulsory treatment centers has been a specific focus of Vietnam’s drug policy. Drug users in Vietnam are reluctant to access existing treatment services, fearing police detection and detention [19].

Limitations of this study include small sample size, convenience sampling, and reliance on self-report. We do not know how many individuals who were asked to participate declined. Our findings may lack generalizability, given the small number of participants from one unique treatment setting. Also, only three out of the nine participants reported history of injection drug use, suggesting on average a less severely addicted population. According to available data, intravenous injection is the most common means of heroin use in China, with injecting drug users accounting for 59–85 % of users [9]. Other potential limitations include issues of translation between English and Mandarin, in which subtle nuances of slang terms and differences in dialects may have limited communication.

## Conclusion

This qualitative study of treatment-seeking heroin users in contemporary China is intended to be exploratory and hypothesis-generating. Our data suggest directions for future research, and raise questions with policy implications, such as:

1. Would launching a public health campaign to decrease the stigma of addiction and promote addiction treatment in China increase utilization of voluntary government-sponsored addiction services?
2. Would eliminating government registration of heroin users increase utilization of voluntary government-sponsored addiction services in China, in particular, methadone maintenance clinics?
3. Would elimination of forced imprisonment when ongoing illicit heroin use is detected, increase utilization of government-sponsored addiction services in China, in particular methadone maintenance clinics?
4. Would reforming forced detention camps in China to include more actual addiction treatment lead to better outcomes, despite their compulsory nature?
5. Do private, for-profit, addiction treatment facilities like New Hospital provide a viable and effective alternative to government-sponsored treatment in China’s evolving drug-treatment landscape?
